# Primary hyperparathyroidism in pregnancy treated with cinacalcet: a case report and review of the literature

**DOI:** 10.1186/s13256-016-1093-2

**Published:** 2016-12-20

**Authors:** Lara Vera, Silvia Oddo, Natascia Di Iorgi, Giorgio Bentivoglio, Massimo Giusti

**Affiliations:** 1 Department of Internal Medicine, Genoa University, Viale Benedetto XV 6, 16132 Genoa, Italy; 2 Department of Pediatrics, IRCC G. Gaslini, Genoa, Italy; 3 Department of Obstetrics and Gynecology, IRCC G. Gaslini, Genoa, Italy

**Keywords:** Cinacalcet, Calcimimetic, Pregnancy, Primary hyperparathyroidism, Hypercalcemia, Case report

## Abstract

**Background:**

The efficacy and safety of various modes of medical treatment for primary hyperparathyroidism in pregnancy are largely unknown.

**Case presentation:**

We report the case of a 34-year-old white woman with primary hyperparathyroidism symptomatic for nephrolithiasis. Her serum calcium was 3.15 mmol/l and parathyroid hormone was 109.0 ng/L. Neck imaging found no pathological parathyroid tissue. Cinacalcet and cholecalciferol were started. She became pregnant 17 months later. The calcimimetic was stopped. During pregnancy, she was admitted for hydration administered intravenously two to three times per week. In her 24^th^ week of pregnancy, cinacalcet was restarted. In her 32nd week, a cesarean section was carried out as planned.

**Conclusions:**

Only three cases of primary hyperparathyroidism in women on cinacalcet therapy in pregnancy have been published in the literature. In the present case, hydration was useful in controlling serum calcium. Cinacalcet therapy helped to control serum calcium.

## Background

Primary hyperparathyroidism (PHPT) is typically a disease of middle-aged and older women. A recent analysis of a large health system database reported the incidence of PHPT to be 4.7 to 6.2 cases per 100,000 person-years in women of reproductive age (20 to 39 years) [1]. PHPT usually runs a relatively benign clinical course. By contrast, during pregnancy PHPT may have serious clinical implications [2], with rates of maternal complications, fetal complications, and fetal/neonatal mortality being estimated at 67 %, 80 % and 30 %, respectively [2, 3]. It is therefore important to recognize this pathology during pregnancy. This is not simple, however, owing to its aspecific presentation [3]. The most frequent complications are hyperemesis, pre-eclampsia, nephrolithiasis, pancreatitis, hypercalcemic crisis, intrauterine growth retardation, preterm labor, neonatal tetany, and neonatal death [2–4]. Although the pregnancy may develop uneventfully, severe fetal/neonatal complications have been reported even in cases of mild PHPT.

The optimal management of PHPT during pregnancy needs to be individualized. Most authors advocate parathyroidectomy (PTx) as the treatment of choice [2]. Surgery should be performed in the second trimester [3]. The safety of surgery in the first and third trimesters is debated because of the associated risks for the fetus [5]. The efficacy and safety of various modes of medical treatment for PHPT in pregnancy are largely unknown. So far, only hydration and calcitonin have emerged as safe treatments [5], although both will lower serum calcium (S-Ca) only temporarily. Bisphosphonates cross the placenta; they should therefore only be used in emergencies as a short-term intervention prior to surgery [6]. Calcimimetics are effective in reducing S-Ca in PHPT; however, they have rarely been used in pregnancy [7].

We describe a case of PHPT in pregnancy that was treated with cinacalcet.

## Case presentation

We report the case of a 34-year-old white woman with a 5-year history of nephrolithiasis, who was referred to the Department of Endocrinology after having undergone a previous endoscopic treatment. A biochemistry evaluation revealed elevated S-Ca and parathyroid hormone (PTH) levels, consistent with severe PHPT. All data from the basic evaluation are reported in Table 1. She had no family history of hypercalcemia or other factors suggestive of multiple endocrine neoplasia (MEN) syndrome. Given her young age, genetic testing was performed, which excluded *MEN1* mutation. On examination, no mass was palpable in her neck. Neck imaging revealed no pathological parathyroid tissue: neck ultrasound, technetium-99m (^99m^Tc) sestamibi scan, and single-photon emission computed tomography-computed tomography (SPECT-CT). Neck ultrasound showed a small nodule of 10 mm in her right thyroid lobe. Her bone density is below the expected range (Z-scores of −1.2 and −1.3 at her spine and total hip, respectively). An abdominal ultrasound confirmed the bilateral presence of kidney stones. Oral hydration, cholecalciferol (600 U/day), vitamin C, and cinacalcet were started, but calcimimetic therapy was poorly tolerated. She became pregnant 17 months later while on therapy.

**Table 1:** Laboratory findings on diagnosis, during pregnancy, and postpartum (median values are reported)

	Diagnosis	Pregnancy	Puerperium	Normal range
Creatinine (mg/dl)	0.7	0.8	0.8	0.5–1.3
GFR (ml/minute/1.73m^2^)	>60.0	107.0	107.0	>60.0
Ca(U) (g/24 hours)	0.46	0.35	–	0.05–0.40
S-Ca (mmol/l)	3.15	3.025	3.0	2.12–2.75
S-P (mg/dl)	1.6	1.9	1.9	2.5–4.5
PTH (ng/l)	109.0	44.0	124.0	6.5–36.5
25OHvitD (ng/ml)	20.1	21.0	13.5	>30.0

*GFR* glomerular filtration rate, *Ca(U)* urinary calcium, *S-Ca* serum calcium, *S-P* serum phosphorous, *PTH* parathyroid hormone, *25OHvitD* 25hydroxyvitamin D

Given the unknown teratogenic effects of cinacalcet in pregnancy, the calcimimetic was discontinued. Cholecalciferol was continued to 1200 U daily. During pregnancy, she presented two to three times per week for saline infusions administered intravenously (sodium chloride, NaCl, 0.9 % 1000 ml two to three times per week) associated with oral hydration at home, but her S-Ca level was not controlled. Conversely, her PTH levels decreased (Table 2). Parathyroid re-exploration was performed in her tenth week of gestation. A nodular area was found at the inferior pole of the left lobe; the location and the sonographic appearance were typical of parathyroid adenoma. Fine-needle washing after aspiration biopsy (PTH-FNAB) was therefore carried out on both nodules. A cytological examination of the left nodules was not diagnostic, but the PTH-FNAB confirmed the thyroidal nature of the nodules: thyroglobulin-fine-needle aspiration biopsy (FNAB) 41334.0 μg/l, calcitonin-FNAB 6.8 ng/l, and PTH-FNAB 4.0 ng/l. The right nodule was a colloid nodule, which was classified according to the British Thyroid Association as Thy2: thyroglobulin-FNAB 23690.0 μg/l, calcitonin-FNAB 6.3 ng/l, and PTH-FNAB 4.0 ng/l. Exploratory surgery was proposed in her second trimester of pregnancy, but was refused and postponed to the postpartum period. Options for medical management were therefore explored. In her 24th week of gestation, daily treatment with cinacalcet (15 to 30 mg/day orally) was restarted, but nausea and hyperemesis ensued. The combination of calcimimetic and intravenously administered hydration resulted in a small decrease in her S-Ca (Table 2). However, in her 25th week, an episode of renal pain due to left nephrolithiasis occurred. Her renal function was normal, as was her electrocardiogram-monitored cardiac function.

**Table 2:** The time-course of serum calcium and parathyroid hormone levels and treatment in a patient with primary hyperparathyroidism

Time	Treatment	Serum calcium (mmol/l)	Parathyroid hormone (ng/L)
1st trimester	NaCl 0.9 % 1000 ml i.v. 2/week	3.025–3.775	48.0
2nd trimester	NaCl 0.9 % 1000 ml i.v. 3/week	2.800–3.325	44.0–62.0
3rd trimester	Cinacalcet 15–30 mg/day	2.775–3.125	–
Postpartum	No therapy	2.900–3.000	46.0–124.0

*i.v.* intravenous, *NaCl* sodium chloride

**Fig. 1:**
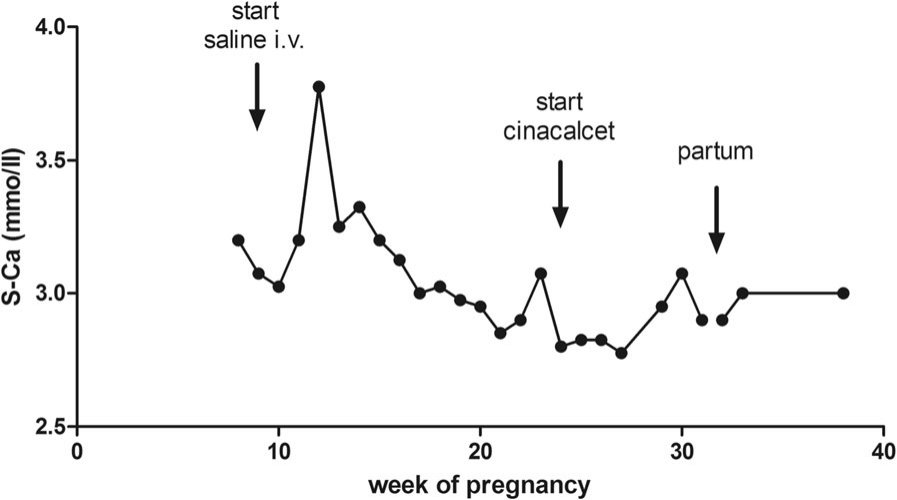
Serum calcium levels in the patient. The graph shows the effect of treatments on the patient’s serum calcium levels during pregnancy and puerperium. *i.v.* intravenous, *S-Ca* serum calcium

She was hospitalized in her 30th week of pregnancy for a non-serious spontaneous rupture of the membranes. A preterm delivery was decided upon. In her 32nd week of pregnancy, a cesarean section was carried out, and a baby boy was delivered. Postpartum, she discontinued cinacalcet in order to start breastfeeding. She was discharged on postoperative day 3, as is normal. A blood test 8 weeks after her cesarean section revealed elevated S-Ca and PTH levels, as noted in her pre-pregnancy period. Breastfeeding was discontinued and cinacalcet was restarted. The time-course of her S-Ca during pregnancy and her puerperium are reported in Fig. 1.

One year after delivery, she underwent surgical exploration of her neck without further localization studies. Ten minutes after surgical removal of her left upper parathyroid gland (1.2 cm), her level of intraoperative PTH was 22 pg/ml. One day after PTx, her S-Ca levels were 2.22 mmol/L. Histology confirmed the diagnosis of adenoma of the parathyroid gland.

### The neonate

**Fig. 2:**
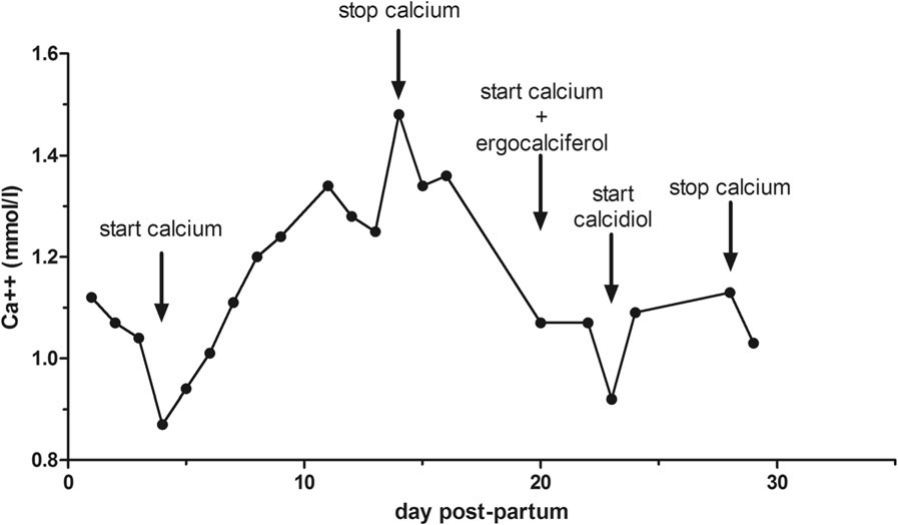
Serum calcium levels in the baby boy. The graph shows the effect of treatments on serum ionized calcium levels in the baby boy in the first 30 days postpartum. *Ca* ^*++*^ ionized calcium

Ultrasound scans in her first and second trimesters revealed normal fetal development. Immediately after delivery, the baby boy (weight 2.03 kg; length 45.0 cm; head circumference 31.7 cm, Apgar score 8 to 9) was transferred to our neonatal intensive care unit. The baby boy had hyperphosphatemia with serum phosphorous (S-P) of 7.94 mmol/l, hypocalcemia with ionized calcium (Ca^++^) of 1.12 mmol/l, and low PTH (17.0 ng/l); a picture compatible with hypoparathyroidism due to maternal hyperparathyroidism. Therefore, oral calcium gluconate 10 % (3 ml×7/day = 100 mg/kg) and vitamin D (ergocalciferol, two drops/day and alfacalcidol, two drops/day) in milk feed were started. A mild episode of neonatal tetany occurred in week 4. After 6 weeks, calcium supplementation was stopped and serum Ca^++^ was stable (Ca^++^ 1.13 mmol/l; S-Ca 3.61 mmol/l). His PTH (38.0 ng/l) and S-P (7.12 mmol/l) were in the normal range. The time-course of Ca^++^ (in the first 30 days postpartum) is reported in Fig. 2.

## Discussion

During pregnancy, the calcium metabolism is altered in order to supply calcium to the fetus. Although S-Ca may decrease owing to lower levels of albumin, Ca^++^ remains constant. In order to meet fetal needs, intestinal calcium absorption doubles under mediation by calcitriol, prolactin, and placental lactogen. There is a compensatory increase in renal calcium excretion, which allows the mother to remain eucalcemic. By contrast, in our case, S-Ca increased considerably, while urinary calcium remained in the normal range, probably owing to a dilution effect induced by forced hydration. Changes in PTH, PTH-related protein (PTHrP), calcitonin, and calcitriol may occur throughout pregnancy, but typically do not produce clinically significant perturbations in calcium balance [8].

Pathologic alterations in calcium metabolism during pregnancy are uncommon, but include PHPT, milk-alkali syndrome or PTHrP-mediated hypercalcemia [9]. During pregnancy and the puerperium, sources of PTHrP include underlying malignancy, placenta, and mammary tissue during lactation: cases have been reported of hypercalcemic crises attributable to each of these sources [10]. Since PTH levels are not proportionately high, the poor control of hypercalcemia may be partly a result of PTHrP. After delivery, hypercalcemia may acutely worsen owing to loss of placental shunting of calcium away from the maternal circulation; alternatively, S-Ca levels may decrease if the placenta was a source of PTHrP, which may have been a factor in our case.

As demonstrated by our patient, PHPT may put the mother and fetus at risk of more severe complications; this case also illustrates the fact that maternal hypercalcemia can cause neonatal hypoparathyroidism, which are manifested in only 12 % of neonates [11].

### Considerations on imaging

The usual techniques for detecting parathyroid adenomas or hyperplasias are not recommended in pregnancy. Technetium (Tc) and 99^m^Tc sestamibi scintigraphy should be avoided, owing to the risk of ionizing radiation for the fetus. The effects of fetal irradiation are divided into deterministic effects (caused by damage to a number of cells in tissues) and stochastic effects (caused by mutations in cells). McMullen *et al*. [12] have described the use of functional imaging in pregnancy while appropriately shielding the fetus; in our case, however, such an approach would have been very unlikely to yield a positive result, as the SPECT-CT performed shortly before the pregnancy was negative. In addition, McMullen *et al*. [12] claimed that 99^m^Tc sestamibi imaging may be used safely to visualize the parathyroid glands only after negative cervical explorations, which our patient had refused. Thus, neck ultrasound is currently the first-line investigation for locating parathyroid diseases during pregnancy (sensitivity of 69 %, specificity of 94 %) [3, 13]. The failure of imaging is most unusual in patients with S-Ca levels higher than 3 mmol/L, as in our case. In our case, an ultrasonography examination was supplemented by PTH-FNAB. The use of this type of examination in the diagnosis of PHPT is debated [14]. In the present case, however, it was necessary in order to tackle the differential diagnosis between PHPT and thyroid nodules and then to decide whether to undertake mini-invasive surgery (recommended) or exploratory surgery (more invasive and therefore not recommended in pregnancy) [12]. Sometimes a mediastinal parathyroid adenoma is the culprit, but pre-pregnancy SPECT-CT had excluded this in our patient.

Finally, computed tomography (CT) and MRI were not performed because when they are used alone they are relatively insensitive in detecting normally located and ectopic parathyroid adenomas [12]. In Italy, positron emission tomography (PET) with methionine has only recently been proposed for the localization of parathyroid glands, and was therefore not performed in our patient before pregnancy.

### Considerations on treatment

Surgery is the first choice of treatment in patients with symptomatic PHPT. Minimally invasive approaches have gained progressive acceptance as an alternative safe and effective technique over the past two decades, owing to the use preoperative imaging to suggest the position of the parathyroid glands [15]. Although negative imaging studies would not preclude neck exploration in a young woman with moderately severe hypercalcemia and recurrent nephrolithiasis, our patient chose to postpone neck exploration with the more invasive techniques required for PTx; medical treatment was therefore initiated. However, bone anti-reabsorptive therapy was not started because her bones had not been significantly compromised.

There are no guidelines for the treatment of PHPT in pregnancy. The options are a conservative approach or surgery [3]. Both approaches carry the risk of drug-related side effects and procedure-related side effects, although the risk imposed by hypercalcemia is sometimes greater. PTx is the only curative treatment, but is recommended only when medical treatment is insufficient [3]. If surgery is deferred, as in our case, it should be undertaken as soon as possible after delivery, to prevent a hypercalcemic crisis.

Conservative management has been shown to significantly reduce maternal and fetal complications [3]. Intravenously or orally administered rehydration, with or without forced diuresis, is the first line of treatment [11]. In our case, we first started hydration without forced diuresis because the patient was hypotensive (her mean arterial pressure was 65 to 70 mmHg). However, as saline therapy administered intravenously is very unlikely to have a lasting benefit in any patient, close maternal and fetal monitoring are necessary in order to prevent clinical or biological deterioration. As calcitonin alone is considered ineffective for long-term S-Ca control, it was not administered in this case. As bisphosphonates are embryotoxic [16], they are not recommended. The use of cinacalcet during pregnancy is debated [3]. However, as S-Ca levels were still very high in our patient, cinacalcet seemed the best choice. Unfortunately, our case seems to indicate that the use of cinacalcet to treat PHPT in pregnancy is not effective in the acute control of S-Ca. In fact, evidence for a true benefit of cinacalcet in our case is very weak. Moreover, we do not know whether this caused a premature decline in S-Ca or compromised fetal calcium metabolism. Before considering cinacalcet treatment, we extensively discussed the pros and cons, as its use in pregnancy has been scantly reported.

### Review of the literature

Since the first case of PHPT in pregnancy was reported in 1932 [4], about 200 cases have been published in the medical literature. Moreover, only three cases of PHPT in women on cinacalcet therapy in pregnancy [8, 17, 18] and two cases in puerperium [17, 19] have been published. Horjus *et al*. [17] first described a case of PHPT, while Edling *et al*. [8] described a case of parathyromatosis and Nadarasa *et al*. [18] reported a case of carcinoma. In the first two cases, therapy was initiated during the third trimester, whereas in our case we decided to start at the end of the second trimester, given the lack of control of S-Ca. In the third case, pregnancy was initiated while the patient was on calcium-lowering therapy, as in our case. In the cases previously reported, tolerance was variable, whereas in our case it was poor, thus precluding administration at higher doses. In our case, unlike those reported in the literature, it was not possible to perform PTx. Therefore, cinacalcet was restarted in the puerperium [17, 19].

## Conclusions

Only three cases of PHPT in women on cinacalcet therapy in pregnancy have been published in the medical literature. Hydration was useful in controlling S-Ca, and cinacalcet therapy also helped to control S-Ca, although it was dangerously high. However, in the present case, evidence for a true benefit of cinacalcet is very weak. In our case, cinacalcet tolerance was very poor, which precluded the administration of higher doses. It remains unclear whether PTH levels in pregnancy were reduced owing to a possible role of placental PTHrP.

## Abbreviations

^99m^Tc: Technetium-99m
Ca^++^: Ionized calcium
CT: Computed tomography
FNAB: Fine-needle aspiration biopsy
MEN: Multiple endocrine neoplasia
NaCl: Sodium chloride
PET: Positron emission tomography
PHPT: Primary hyperparathyroidism
PTH: Parathyroid hormone
PTH-FNAB: Fine-needle washing after aspiration biopsy
PTHrP: Parathyroid hormone-related protein
PTx: Parathyroidectomy
S-Ca: Serum calcium
S-P: Serum phosphorous
SPECT-CT: Single-photon emission computed tomography-computed tomography
Tc: Technetium
